# A mucoadhesive, thermoreversible *in situ* nasal gel of geniposide for neurodegenerative diseases

**DOI:** 10.1371/journal.pone.0189478

**Published:** 2017-12-14

**Authors:** Yingting Wang, Shulong Jiang, Hongli Wang, Haiyan Bie

**Affiliations:** Jining No.1 People's Hospital, Jining, Shandong Province, China; Brandeis University, UNITED STATES

## Abstract

Neurodegenerative diseases are becoming prevalent as the population ages. Geniposide could inhibit oxidative stress, reduce apoptosis, protect neuron, and has been used for therapy of the neurodegenerative diseases. The bioavailability of geniposide by nasal route is greater than that by oral administration. However, mucociliary clearance is a rate-limiting factor for nasal route administration. The objective of this study was to develop and evaluate a mucoadhesive, thermoreversible *in situ* nasal gel of geniposide. The poloxamers (P407, P188) and the hydroxypropyl methylcellulose were used as thermoreversible and mucoadhesive polymers, respectively. Borneol was used as a permeation enhancer. The hydrogel was prepared with the cold method and optimized by the response surface methodology-central composite design. Gelation temperature, pH, clarity, gel strength, mucoadhesive strength, *in vitro* and *ex vivo* release kinetics of formulations were evaluated. The optimized amounts of poloxamer407 (P407), poloxamer188 (P188) and hydroxypropyl methylcellulose were determined to be 19.4–20.5%, 1.1–4.0% and 0.3–0.6% respectively. The second-order polynomial equation in terms of actual factors indicated a satisfactory correlation between the independent variables and the response (*R*^2^ = 0.9760). An ANOVA of the empirical second-order polynomial model indicated the model was significant (*P*<0.01). P407, P188, P407×P188, P407^2^ and P188^2^ were significant model terms. The effects of P407 on gelation temperature were greater than those of other independent variables. The pH values of all the formulations were found to be within 6.3–6.5 which was in the nasal physiological pH range 4.5–6.5. The drug content, gel strength, mucoadhesive strength of the optimized formulations were 97–101%, 25–50 sec and 4000–6000 dyn/cm^2^ respectively. The *in vitro* release kinetics of cumulative release of geniposide was fitted to the zero-order model. The *ex vivo* cumulative release kinetics of geniposide was fitted to the Weibull model. This study concludes that the release of geniposide is controlled by gel corrosion, and that the permeation of geniposide is time-dependent. The more residence time, mucoadhesive, thermoreversible *in situ* nasal gel of geniposide for neurodegenerative diseases is of compliance and potential application.

## Introduction

Neurodegenerative diseases are becoming prevalent as the population ages. The neurodegenerative diseases include Alzheimer’s disease (AD), Parkinson’s disease (PD), amyotrophic lateral sclerosis (ALS), frontotemporal dementia (FTD) and so on [1–4]. Geniposide is a pharmacologically active compound in *Gardenia jasminoides* Ellis (Rubiaceae) used for the treatment of hepatic disease, inflammation disorders, contusions and brain disorders [5–7]. Accumulated research data showed geniposide could inhibit oxidative stress and mitochondrial dysfunction [8], improve cognition [9], inhibit the interaction between amyloid-beta peptide and RAGE, attenuate Aβ-induced neuronal injury [10,11]. The order of absolute bioavailability of geniposide was oral (F = 9.74%) < nasal drops (F = 49.54%) < intramuscular (F = 72.69%). The nasal route delivered geniposide to brain directly through the olfactory region [12]. The pharmacokinetics parameters of intranasal (i.n.) and intragastric (i.g.) administration were compared with those of intravenous (i.v.) administration. The bioavailabilities of geniposide were 85.38% (i.n.) and 28.76% (i.g.) [13]. Nasal delivery of drugs could improve better patient compliance than intravenous (i.v) administration [14]. The nasal route has been considered as a viable and efficacious alternative for drugs which have extensive first pass metabolism [15]. One of the major disadvantages to deliver drugs through nasal route is the mucociliary clearance [16]. To address this issue, mucoadhesive *in situ* gel formulation was devised to increase the residence time in the nasal cavity [17]. The bioavailability of geniposide might be promoted if the drug remains longer time inside the nasal cavity. The borneol could increase transportation of geniposide across the human nasal epithelial cell [18, 19]. Poloxamer was temperature-triggered, nontoxic, nonirritating and non-sensitizing polymer [20–22]. Poloxamer and hydroxypropyl methylcellulose have been used for the *in situ* gel [23–25]. Until now, mucoadhesive, thermoreversible *in situ* nasal gel of geniposide has not been reported.

Taking the above factors into consideration, the present study developed and evaluated a more residence time, mucoadhesive, thermoreversible *in situ* nasal gel of geniposide for the neurodegenerative diseases.

## Materials and methods

### Materials

Geniposide (95.0% purity) and borneol (86.3% purity) were provided by the First People’s Hospital in Jining. Geniposide standard (97.5% purity) was purchased from the National Institute for Food and Drug Control (Beijing, China). Poloxamers (P407, P188) were purchased from Sinopharm Chemical Reagent Co., Ltd (Shanghai, China). Hydroxypropyl methylcellulose (HPMC K4M) was purchased from Anhui Sunhere Pharmaceutical Excipients Co., Ltd (Anhui, China). Acetonitrile (HPLC grade) was purchased from Avantor Performance Materials Trading Co., Ltd (Shanghai, China). Benzalkonium chloride (BC) and sodium chloride were purchased from Sinopharm Chemical Reagent Co., Ltd (Shanghai, China). All other reagents were of analytical grade.

### Determination of geniposide

Geniposide was quantified by high performance liquid chromatography equipped with Waters e2695 separation module and 2998 photodiode array detector (Waters, the United States). The chromatographic separation was achieved using a Waters X-Bridge C18 column (5μm, 4.6 mm × 250 mm). The mobile phase was an acetonitrile: purified water (15: 85) mixture. The detector was set 238 nm. The column temperature was controlled 25°C. The volume of each injection was 20μl.

### Preparation of formulation

The cold method was adopted for preparing formulation [26–28]. First of all, purified water was stored overnight at 4°C in refrigerator. Secondly, the required amount of poloxamer (P407 and P188) was slowly added into the required volume of cold purified water with continuous stirring, and then the dispersion was kept overnight at 4°C until a transparent hydrogel was obtained. Thirdly, the required amount of hydroxypropyl methylcellulose, benzalkonium chloride (0.001%, w/v) and sodium chloride (0.9%, w/v) were dispersed into the hydrogel with continuous stirring. Finally, geniposide and borneol were added into the upper solution. The volume was adjusted, and then stored in refrigerator again until transparent formulations were prepared.

### Clarity of formulation

Formulation was observed visually under black and white background. The clarity of formulation was graded as follows: turbid: +, clear: ++, very clear (glassy): +++ [29].

### pH of formulation

The pH value of formulation was determined by using the pH meter (HANNA, P211). The pH meter was first calibrated using solutions of pH 7.01 and pH 4.01.

### Gelation temperature of formulation

The gelation temperature of formulation was determined by the tube inversion method as reported previously [30, 31]. Briefly, the hydrogel (0.5 ml) was transferred to small vial (2 ml, 12 mm × 32 mm), which was sealed and immersed into a thermostat controlled-electric water bath at an initial temperature of 20°C. The temperature of the water bath was increased in increment of 0.5°C/min. The mercury bulb of a thermometer with a minimum readable scale of 0.2°C was placed at the same level with the hydrogel. The meniscus of the hydrogel didn’t move when the vial was tilted 90 degree angle. The temperature on the thermometer was identified as the gelation temperature. The gelation temperature was recorded and measured in triplicate.

### Gel strength of formulation

The gel strength of formulation was determined as reported previously [32–34]. A sample of 50 g of hydrogel was put into a 100 ml graduated cylinder and gelled in a thermostatically controlled water bath at 37±0.5°C. A weight of 35 g was placed onto the gel. The gel strength, which was an indication of viscosity, was determined by the time in second required by the weight to penetrate 5 cm into the gel.

### Mucoadhesive strength of formulation

The mucoadhesive strength of formulation was determined as reported previously [35, 36]. A section of goat nasal mucosa was obtained from local slaughter house immediately after its sacrificesacrifice. Two cylindrical glass vials with 2 cm diameter and modified balance instrument were taken. The goat nasal mucosa was tied to one side of the both vials. Fifty milligrams of hydrogel was placed on one nasal mucosa of one vial. The two vials’ nasal mucosa were attached together for 2 min. Water was poured drop by drop into the container of the balance instrument until the two vials got detached from each other. The water was weighed. The mucoadhesive strength of formulation was expressed as the detachment stress in dyne/cm^2,^ and was determined by minimal.

$$\begin{array}{l} \mathrm{Mucoadhesive}\;\mathrm{strength}\;\left(\mathrm{dyne}/cm^{2}\right)=m\times g/A\;(m=\mathrm{Weight}\;\mathrm{required}\;\mathrm{for}\\ \mathrm{detachment}\;\mathrm{of}\;\mathrm{two}\;\mathrm{vials}\;\mathrm{in}\;\mathrm{grams},\;g=\mathrm{Acceleration}\;\mathrm{due}\;\mathrm{to}\;\mathrm{gravity}\;\left(980\;\mathrm{cm}/s^{2}\right),\\ \;A=\mathrm{The}\;\mathrm{area}\;\mathrm{of}\;\mathrm{nasal}\;\mathrm{mucosa}\;\mathrm{exposed}). \end{array}$$

### *In vitro* release kinetics of formulation

*In vitro* corrosion of *in situ* gel and the release of geniposide from the *in situ* gel were studied simultaneously through a membraneless method as reported previously [37–39]. The membraneless model allowed the release medium to directly contact the gels surface. The cold hydrogel (5 g) was transferred into a graduated test tube (1 cm diameter), which was placed in water bath (34±0.5°C) and maintained 10 min. A saline phosphate buffer (pH = 6.4, 2.5 ml) used as release medium, pre-equilibrated at 37±0.5°C, was layered over the surface of the gel. After removing the medium at predetermined one-hour interval, the test tube was cleared, weighted and layered with fresh saline phosphate buffer (2.5 ml). The repeated test procedure was finished until less than 10% of the gel was remained. The amount of geniposide in the samples was determined by the high-performance liquid chromatography method. The experiment was performed in triplicate.

$$\begin{array}{l} \mathrm{Cumulative}\;\mathrm{gel}\;\mathrm{dissolved}\;\mathrm{rate}=\mathrm{cumulative}\;\mathrm{gel}\;\mathrm{dissolved}/\mathrm{initial}\;\mathrm{gel}\times 100\%\\ \mathrm{Cumulative}\;\mathrm{geniposide}\;\mathrm{released}\;\mathrm{rate}=\mathrm{cumulative}\;\mathrm{geniposide}\;\mathrm{released}/\mathrm{initial}\;\mathrm{geniposide}\times 100\% \end{array}$$

### *Ex vivo* drug permeation

*Ex vivo* drug permeation was studied as previously reported [40–42]. Nasal cavity of goat was obtained from local slaughter house. It was safely transported to laboratory by keeping it in the saline phosphate buffer (pH6.4). The intact nasal mucosa was separated, cleaned and stored in the saline phosphate buffer. The study was conducted using a Franz diffusion system (RYJ-12B, Shanghai China). The nasal mucosa was fixed on the Franz diffusion cell having effective permeation area of 2.8 cm^2^. After 30 min of incubation time, the optimized formulation 0.5 g was placed in the donor compartment. The temperature of the chamber was maintained at 34±0.5°C. The saline phosphate buffer (pH6.4, 6.5 ml) was used as receptor medium. Receptor medium 1 ml was withdrawn from the receptor chamber at the predetermined 30 min interval, and immediately replaced by the fresh saline phosphate buffer maintained at 34±0.5°C. The geniposide of sample was determined by the high- performance liquid chromatography method. The experiment was performed in triplicate. The *ex vivo* geniposide permeation data were evaluated in different mathematical models.

http://dx.doi.org/10.17504/protocols.io.ks2cwge.

## Results

### Pre-formulation on gelation temperature

As shown in Table 1, the poloxamer exhibited the phenomenon of reverse thermal gelling under a certain concentration and temperature. The formulation with P407(16.0–24.0%) and P188 (1.0–8.0%) formed a semisolid transparent gel at a certain temperature. The gelation temperature decreased gradually as the P407 concentration increased. However, the temperature increased as the P188 concentration increased. Therefore, P188 was used in combination with P407 to regulate the gelation temperature for more suitable gel formulations.

**Table 1:** Effects of P188 and P407 concentration on the gelation temperature (n = 3, °C).

P188(%, w/v)	P407 (%, w/v)				
	16.0	18.0	20.0	22.0	24.0
0	40.1±0.2	34.5±0.2	28.6±0.1	23.4±0.1	18.0±0.3
1.0	42.1±0.1	35.3±0.1	30.1±0.2	26.2±0.1	21.9±0.2
2.0	46.3±0.3	37.1±0.1	34.6±0.2	28.9±0.1	23.7±0.1
4.0	48.3±0.3	39.6±0.1	36.6±0.3	32.0±0.1	26.5±0.1
6.0	49.6±0.2	42.7±0.2	40.8±0.2	33.3±0.1	29.8±0.1
8.0	51.3±0.2	44.8±0.2	44.1±0.3	36.6±0.2	32.3±0.3

### Effects of additives and geniposide on gelation temperature

Drug and additives changed the gelation temperature (Table 2). The gelation tempera-ture decreased from 33.2°C to 30.3°C as the hydroxypropyl methylcellulose (HPMC) concentration increased from 0.1% to 1.0% (F3-F5). Benzalkonium chloride (BC) and sodium chloride (NaCl) decreased gelation temperature from 36.6°C to 33.8°C (F1 vs F2). However, the addition of the geniposide and borneol increased gelation temperature from 33.8°C to 35.2°C (F2 vs F6). The amounts of P407, P188 and HPMC should be optimized to achieve a more suitable gelation temperature for the thermoreversible *in situ* nasal gel of geniposide. All the formulations were transparent.

**Table 2:** Effects of BC, NaCl, HPMC, geniposide and borneol on gelation temperature (n = 3).

	Concentration (w/v)	Clarify	pH	Tgel (°C)
F1	P407 20%+P188 4%	+++	6.3	36.6±0.3
F2	P407 20%+P188 4%+ BC 0.001% + NaCl 0.9%	+++	6.4	33.8±0.2
F3	P407 20%+P188 4%+ BC 0.001% + NaCl 0.9% +HPMC 0.1%	++	6.2	33.2±0.1
F4	P407 20%+P188 4%+ BC 0.001% + NaCl 0.9% +HPMC 0.5%	++	6.1	32.7±0.1
F5	P407 20%+P188 4%+ BC 0.001% + NaCl 0.9% +HPMC 1.0%	++	6.2	30.3±0.2
F6	P407 20%+P188 4%+ BC 0.001% + NaCl 0.9% +geniposide 1.0%+ borneol 1.1%	++	6.5	35.2±0.2

Note: turbid: +, clear: ++, very clear (glassy): +++

### Optimization of formulation

P407 (18.0–24.0%), P188 (1.0–8.0%) and HPMC (0.1–1.0%) were the independent variables. Gelation temperature was the response variable. A three-factor and five-level (-Alpha, -1, 0, 1, +Alpha) full factorial design was employed for the optimization of *in situ* gel of formulations geniposide (Table 3).

**Table 3:** Independent variables and natural levels.

Independent variables	Levels and ranges				
	-α	low	medium	high	+α
	-1.6818	-1.0	0	+1.0	+1.6818
P407 (%)	18.0	19.2	21.0	22.8	24.0
P188 (%)	1.0	2.4	4.5	6.6	8.0
HPMC (%)	0.1	0.3	0.6	0.8	1.0

**Fig 1:**
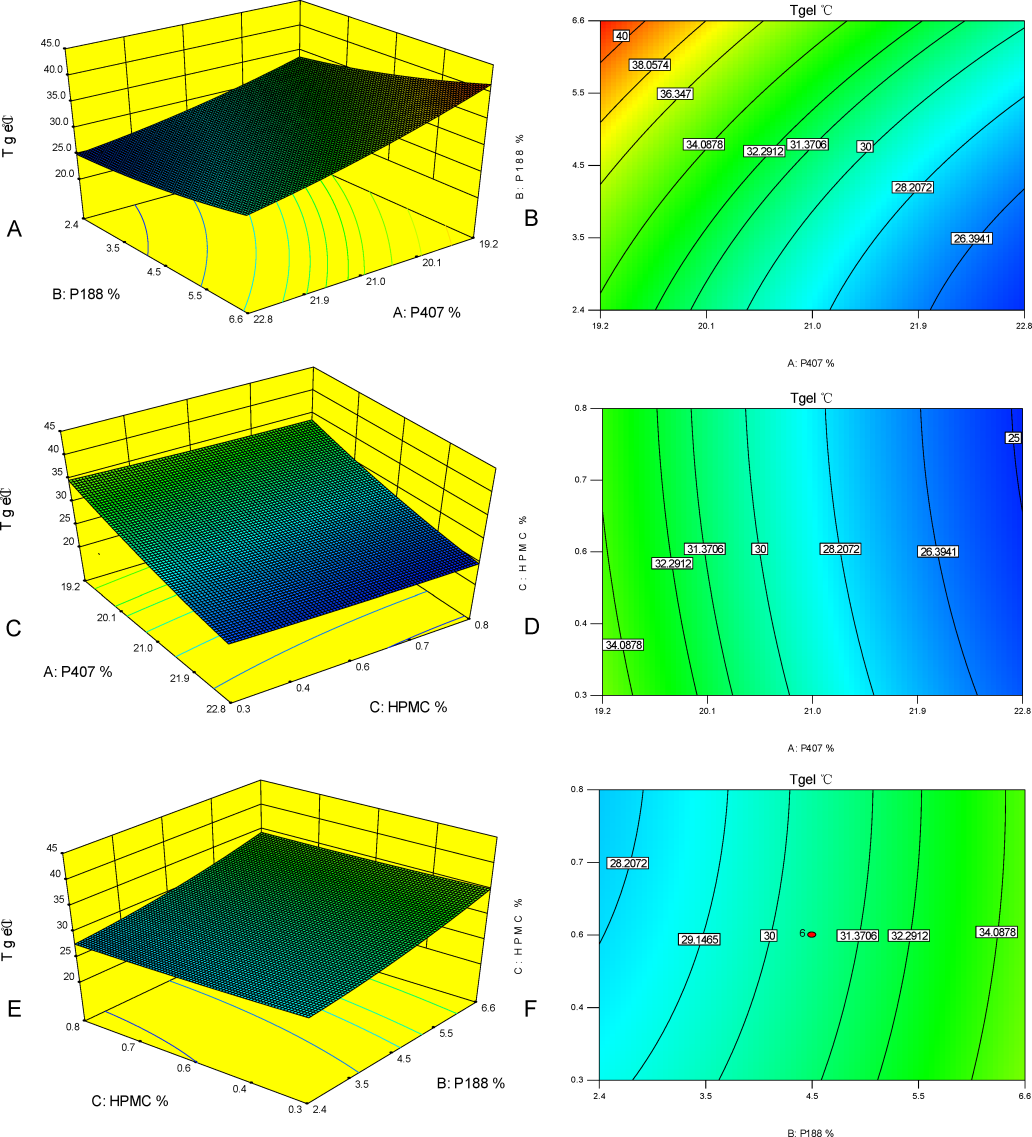
Three-dimensional surfaces and contours showing the influences of P407, P188 and HPMC. (A) 3D surface plot of P407 and P188 on Tgel (HPMC = 0.4%). (B) Contour of P407 and P188 on Tgel (HPMC = 0.4%). (C) 3D surface plot of P407 and HPMC on Tgel (P188 = 3.1%). (D) Contour of P407 and HPMC on Tgel (P188 = 3.1%). (E) 3D surface plot of P188 and HPMC on Tgel (P407 = 21.0%). (F) Contour of P188 and HPMC on Tgel (P407 = 21.0%).

Central composite design (CCD) was employed to evaluate the influence of the three independent variables in runs of 20 experiments. As shown in Table 4, based on the experimental results, a second-order polynomial equation using actual values demonstrated the empirical relationships between the independent variables and the response as follows:

Tgel = 192.7753–13.4382×P407+3.2916×P188-8.7255×HPMC-0.1687×P407×P188+ 0.1389×P407×HPMC+0.1667×P188×HPMC+0.2702×P407^2^+0.1905×P188^2^+2.9784 ×HPMC^2^ (*R*^*2*^ = 0.9760). Regression analysis with a *R*^2^ value 0.9760 indicated a satisfactory correlation between the independent variables and the response. Analysis of variance (ANOVA) was applied to evaluate the adequacy of the empirical second- order polynomial model (Table 5), the Model *F*-value of 45.1780 (*P*<0.0001) implied the model was significant. In the present study, P407, P188, P407×P188, P407^2^ and P188^2^ were significant model terms. Three-dimensional surfaces and contours (Fig 1) as graphical representations of the regression equation showed the considerable influences of P407, P188 and HPMC on the gelation temperature. Gelation temperature increased smoothly with the increase of P188 from 1.0% to 8.0%.Gelation temperature obviously decreased with the increase of P407 from 18.0% to 24.0%. Effect of HPMC on gelation temperature was slight.

**Table 4:** Central composite design experiments and experimental results.

Runs	Independent variables in coded form			Independent variables in their natural form			Tgel value (°C)	
	407 (%)	188 (%)	HPMC (%)	407 (%)	188 (%)	HPMC (%)	Predicted	Actual
1	1.0	1.0	-1.0	22.8	6.6	0.3	30.4	29.4±0.1
2	-1.0	1.0	-1.0	19.2	6.6	0.3	41.8	41.9±0.3
3	0.0	0.0	-1.6818	21.0	4.5	0.1	31.9	31.8±0.2
4	1.0	-1.0	1.0	22.8	2.4	0.8	24.4	24.3±0.3
5	0.0	0.0	1.6818	21.0	4.5	1.0	30.4	30.5±0.2
6	1.0	-1.0	-1.0	22.8	2.4	0.3	25.4	25.2±0.1
7	1.0	1.0	1.0	22.8	6.6	0.8	29.9	28.6±0.3
8	-1.6818	0.0	0.0	18.0	4.5	0.6	41.5	40.0±0.2
9	-1.0	1.0	1.0	19.2	6.6	0.8	41.0	41.1±0.3
10	-1.0	-1.0	1.0	19.2	2.4	0.8	33.0	34.0±0.2
11	1.6818	0.0	0.0	24.0	4.5	0.6	24.7	26.2±0.1
12	0.0	1.6818	0.0	21.0	8.0	0.6	38.5	39.7±0.3
13	-1.0	-1.0	-1.0	19.2	2.4	0.3	34.2	35.4±0.2
14	0.0	-1.6818	0.0	21.0	1.0	0.6	27.5	26.3±0.1
15~20	0.0	0.0	0.0	21.0	4.5	0.6	30.6	30.6±0.1

**Table 5:** ANOVA for response surface quadratic model.

Source	Sum of squares	df	Mean Square	*F*-value	*P*-value
Model	510.9341	9	56.7705	45.1780	< 0.0001
A-P407 (%)	339.6683	1	339.6683	270.3087	< 0.0001
B-P188 (%)	145.8883	1	145.8883	116.0981	< 0.0001
C-HPMC (%)	2.7124	1	2.7124	2.1586	0.1725
AB	3.2512	1	3.2512	2.5874	0.0188
AC	0.0313	1	0.0313	0.0249	0.8778
BC	0.0613	1	0.0613	0.0487	0.8297
A^2^	11.0482	1	11.0482	8.7922	0.0142
B^2^	10.1740	1	10.1740	8.0965	0.0174
C^2^	0.4994	1	0.4994	0.3974	0.5426

### Gel strength and mucoadhesive strength of the optimized formulations

Eight groups of formulations which met the criteria (Table 6) were listed (Table 7). Clarity, pH, gelation temperature, gel strength and mucoadhesive strength of the optimized formulations were evaluated gradually (Table 7). All the optimized formulations were transparent. The pH values of all the formulations were found to be within 6.3–6.5 which was in the nasal physiological pH range 4.5–6.5. Gelation temperature values of the optimized formulations were 29.6–31.3°C, which were in the nasal physiological temperature range 29–34°C. The gel strength values of the optimized formulations were between 29 sec and 67 sec. Mucoadhesive strength values of the optimized formulations were between 3885 dyn/cm^2^ and 1 0935 dyn/cm^2^.

**Table 6:** Criteria for the optimized formulations.

Name	Goal values	Lower limit	Upper limit
P407 (%)	in range	18.0	22.8
P188 (%)	in range	1.0	8.0
HPMC (%)	in range	0.1	1.0
Tgel (°C)	31.0	30.0	32.0

**Table 7:** Parameters of the optimized formulations (n = 3).

F	P407 (%)	P188 (%)	HPMC (%)	pH	Clarity	Tgel (°C)	Gel strength (sec)	Mucoadhesive strength (dyn/cm^2^)
S1	21.1	4.0	0.1	6.3	++	30.6±0.2	29±1.7	4560±106
S2	20.2	1.5	0.2	6.4	++	30.8±0.2	35±2.4	3885±68
S3	20.5	3.3	0.3	6.5	++	30.8±0.1	37±2.5	4450±46
S4	19.7	1.3	0.5	6.4	++	29.7±0.2	38±0.9	4555±82
S5	19.9	2.4	0.6	6.4	++	30.2±0.1	46±1.2	5330±53
S6	19.6	1.5	0.8	6.3	++	31.0±0.3	58±1.8	6780±72
S7	19.6	1.6	0.9	6.5	++	31.3±0.2	59±1.2	8945±115
S8	19.4	1.1	1.0	6.5	++	29.6±0.3	67±2.2	10935±62

Note: turbid: +, clear: ++, very clear (glassy): +++

### *In vitro* release kinetics of geniposide

**Fig 2:**
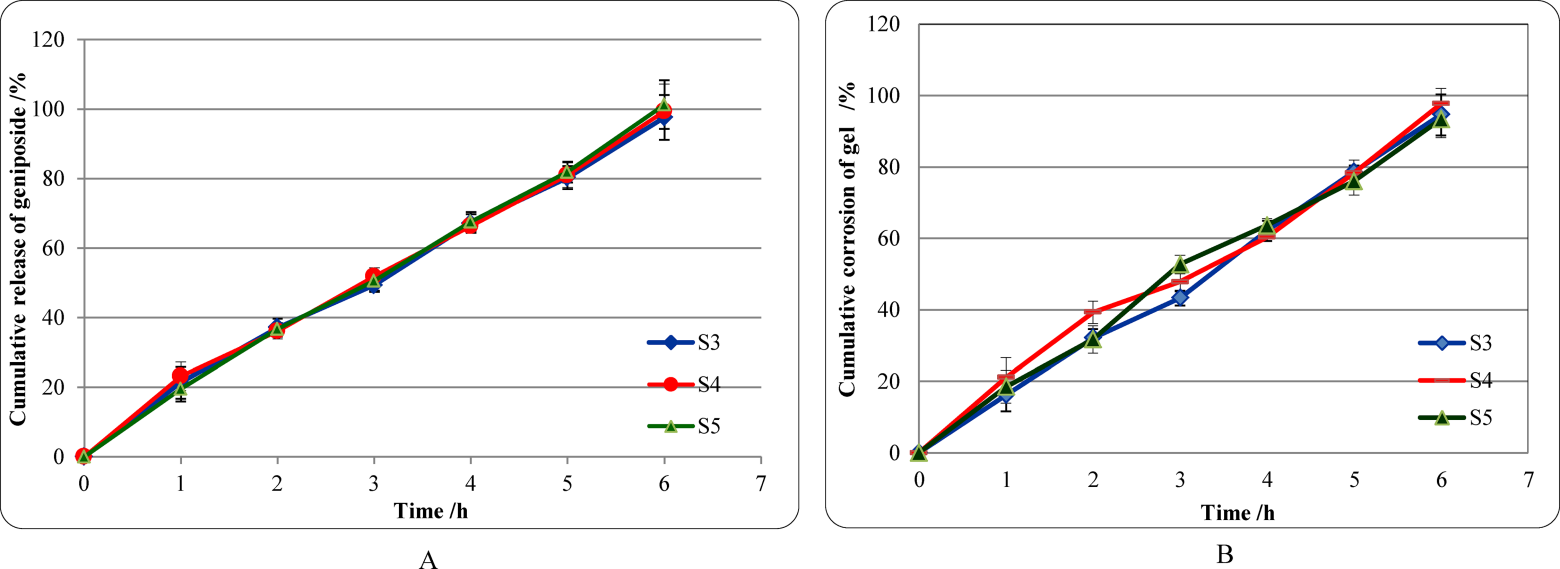
The release features of formulation *in vitro*. (A) Cumulative release of geniposide from the optimized formulations. (B) Cumulative corrosion of gel of the optimized formulations.

**Fig 3:**
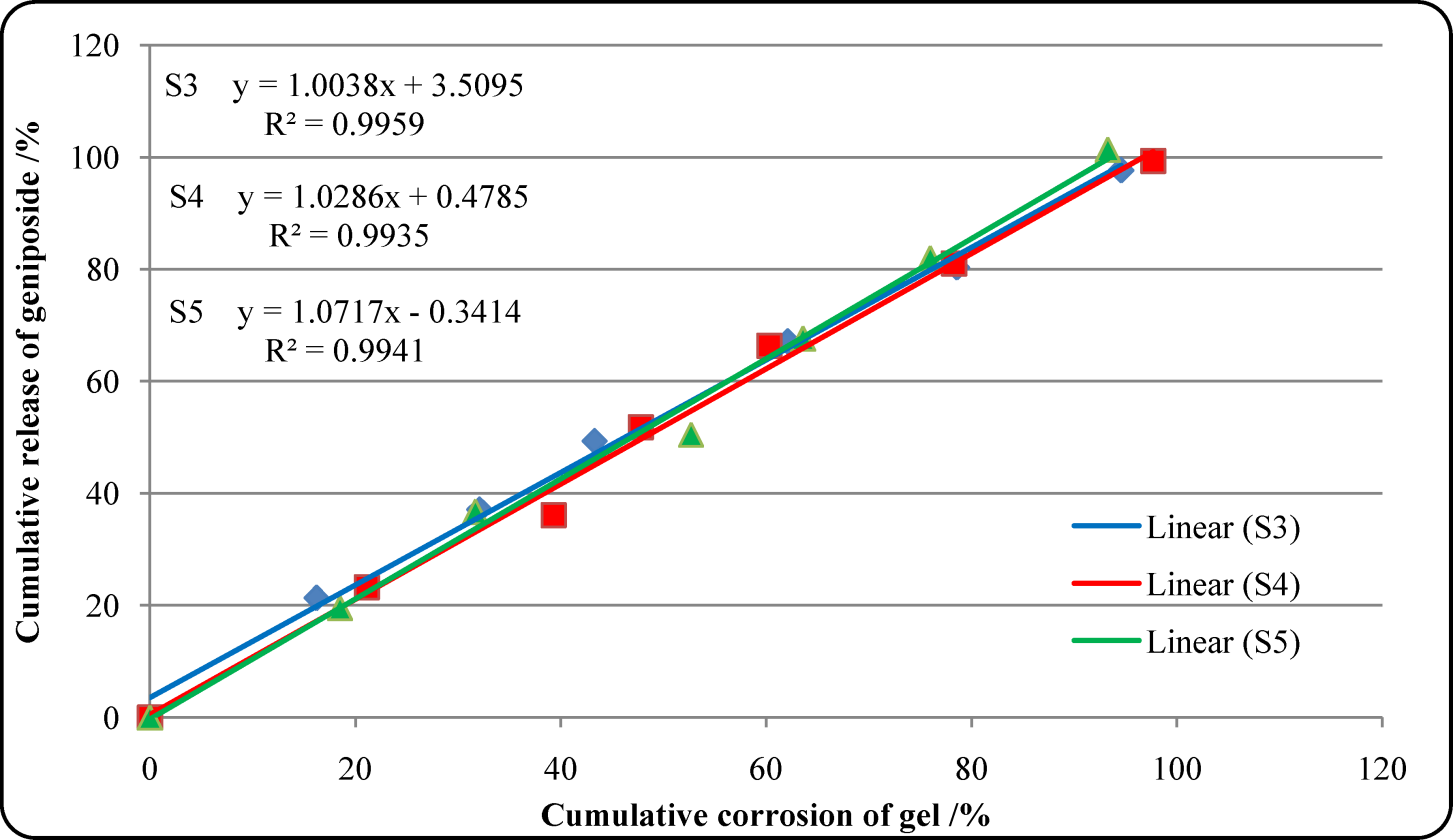
Release kinetics of geniposide from the optimized formulations.

**Fig 4:**
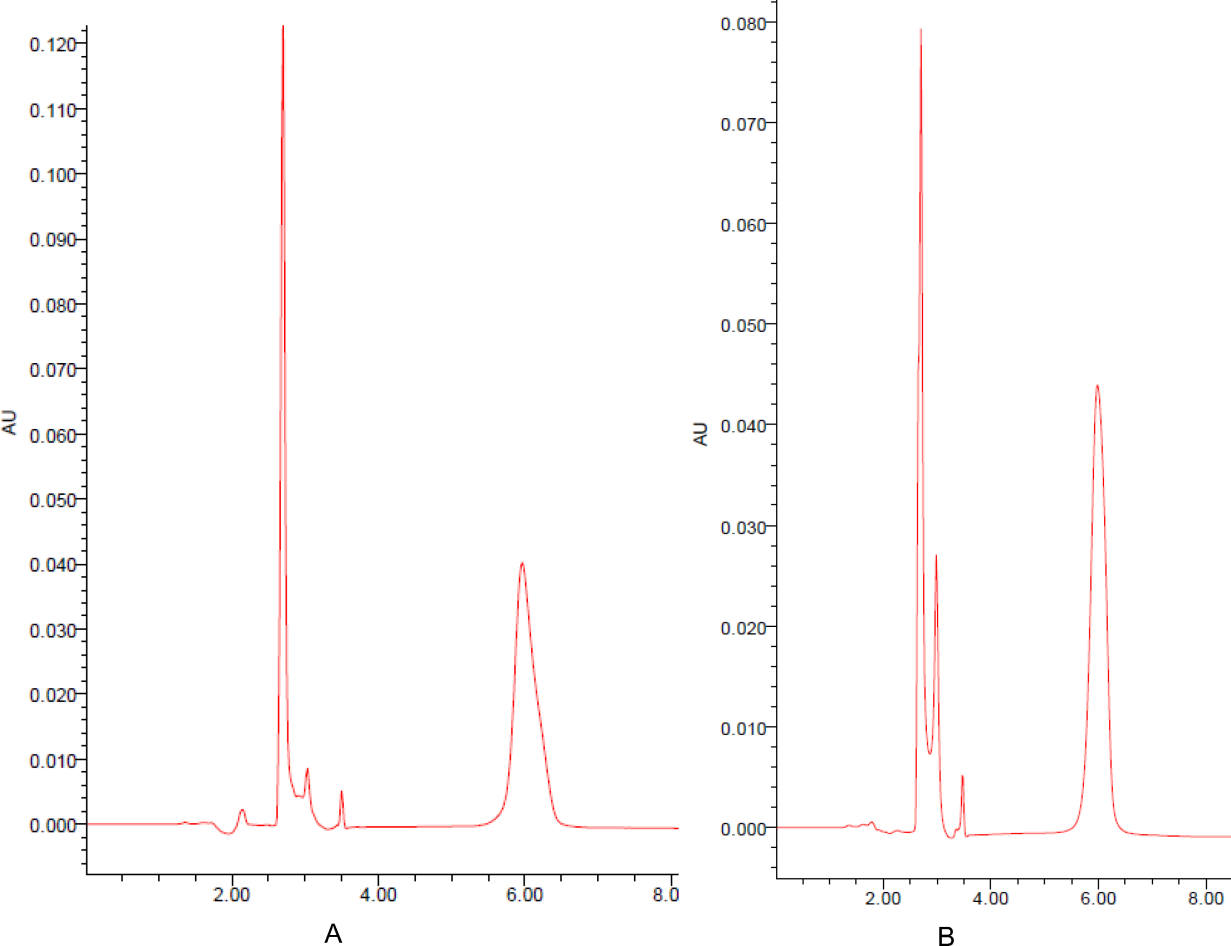
Determination by HPLC method. (A) Representative HPLC chromatogram of geniposide (5.967 min). (B) Chromatogram of geniposide of the optimized formulations (5.990 min).

As shown in Fig 2 and Table 8, the cumulative release rate of the geniposide from the optimized formulations (S3, S4, S5) versus time increased slowly without burst release condition, and the average rate was 99.4% after 6 h. The cumulative corrosion average rate of gel was 95.2% after 6 h. To understand the release mechanism of geniposide from *in situ* gel, data were analyzed with DD Solver (1.0) software. The order of suitable model was Zero-order model > Weibull model >First-order model > Higuchi model (Fig 3 and Table 9). A representative HPLC chromatogram of geniposide and a chromatogram of geniposide of the optimized formulations were presented in Fig 4.

**Table 8:** Corrosion of gel and release of geniposide from the optimized formulations.

Time (h)	Cumulative corrosion of gel (%)			Cumulative release of geniposide (%)		
	S3	S4	S5	S3	S4	S5
1	16.2±4.6	21.1±5.6	18.5±4.6	21.3±4.6	23.2±4.1	19.5±3.6
2	32.1±2.5	39.3±3.1	31.7±3.9	37.1±2.6	36.1±2.2	36.7±1.8
3	43.3±2.1	47.8±2.8	52.7±2.6	49.3±1.9	51.8±2.6	50.5±2.8
4	62.1±2.8	60.3±1.2	63.6±1.9	67.1±2.7	66.3±1.7	67.6±2.8
5	78.6±1.9	78.3±3.6	76.0±3.9	80.3±3.3	81.0±3.6	81.9±3.0
6	94.6±5.8	97.7±4.3	93.3±5.1	97.6±6.5	99.3±8.0	101.3±7.0

**Table 9:** *In vitro* release kinetics models of geniposide from the optimized formulations.

Model	Zero-order	First-order	Higuchi	Weibull
S3	Y = 3.512+1.003*X Rsqr = 0.9959	Y = 100*[1-Exp(-0.018*X)] Rsqr = 0.9493	Y = 8.597*X^0.5 Rsqr = 0.9171	Y = 100*{1-Exp[-(X^1.449)/324.419]} Rsqr = 0.9835
S4	Y = 0.480+1.029*X Rsqr = 0.9935	Y = 100*[1-Exp(-0.017*X)] Rsqr = 0.9132	Y = 8.430*X^0.5 Rsqr = 0.8782	Y = 100*{1-Exp[-(X^1.765)/1250.221]} Rsqr = 0.9827
S5	Y = 1.072*X-0.339 Rsqr = 0.9941	Y = 100*[1-Exp(-0.018*X)] Rsqr = 0.9084	Y = 8.602*X^0.5 Rsqr = 0.8721	Y = 100*{1-Exp[-(X^1.661)/797.704]} Rsqr = 0.9660

Note: X: Cumulative corrosion of gel (%). Y: Cumulative release of geniposide (%).

### *Ex vivo* permeation kinetics of geniposide

As shown in Table 10, the cumulative geniposide permeation rates of the optimized formulations were 83.1–86.8% after 6 h. To understand the release and permeation mechanism of geniposide, data were analyzed with DD Solver (1.0) software. The order of suitable model was Weibull model > Zero-order model > First-order model > Higuchi model (Table 11).

**Table 10:** *Ex vivo* cumulative permeation rate of geniposide from optimized formulations.

Time (h)	S3	S4	S5
0.5	8.1±1.2	7.9±2.3	11.2±1.0
1.0	16.8±1.9	18.6±2.3	16.3±2.1
1.5	25.3±3.2	26.9±1.6	23.9±3.2
2.0	33.7±2.8	38.1±3.7	35.2±3.3
2.5	46.1±3.1	45.6±5.5	43.3±2.4
3.0	53.5±1.6	55.5±3.1	50.5±4.6
3.5	61.3±5.6	63.6±3.4	61.1±3.8
4.0	74.1±2.1	73.0±3.0	67.4±4.2
4.5	79.5±3.6	77.4±1.7	72.8±3.6
5.0	82.6±2.1	83.1±1.6	79.1±1.0
5.5	84.6±6.3	86.3±5.6	80.6±5.6
6.0	86.8±5.3	89.8±4.6	83.1±7.0

**Table 11:** *Ex vivo* permeation kinetics models of geniposide from the optimized formulations.

Model	Zero-order	First-order	Higuchi	Weibull
S3	Y = 4.442+15.369*t Rsqr = 0.9678	Y = 100*[1-Exp(-0.282*t)] Rsqr = 0.9423	Y = 50.553*t^0.5–37.762 Rsqr = 0.9824	Y = 100*{1-Exp[-t^1.457)/6.159]} Rsqr = 0.9943
S4	Y = 5.702+15.317*t Rsqr = 0.9771	Y = 100*[1-Exp(-0.290*t)] Rsqr = 0.9532	Y = 50.410*t^0.5–31.3999 Rsqr = 0.9928	Y = 100*{1-Exp[-(t^1.408)/5.600]} Rsqr = 0.9979
S5	Y = 5.976+14.174*t Rsqr = 0.9757	Y = 100*[1-Exp(-0.260*t)] Rsqr = 0.9621	Y = 46.469*t^0.5–28.049 Rsqr = 0.9839	Y = 100*{1-Exp[-(t^1.327)/5.763]} Rsqr = 0.9947

Note: Y: Cumulative release of geniposide (%). t: Time (h)

## Discussion

Nasal drug delivery has received a significant attention as a convenient and reliable route for local administration of drugs [43, 44]. The nasal cavity offered a distinctive advantage for potential direct drug delivery to the brain along the olfactory nerves [34, 45, 46]. The rapid mucociliary clearance was important rate-limiting factor for nasal drug absorption [15, 47]. For the reason, researches have oriented toward the application of the bioadhesive polymers to extend formulations’ residence time in the nasal cavity for better drug bioavailability [48, 49]. *In situ* forming polymeric formulation was the ideal drug delivery for nasal drops [50]. In this study, the poloxamer407 and the poloxamer188 were used as the basic excipients. The ideal formulation and gelation temperature were got by optimizing proportion of poloxamer407 and poloxamer188.

Determination of gelation temperature is a major step in the preparation of the thermoreversible gel [51, 52]. The thermoreversible hydrogel for nasal drug delivery should be gelled in 25–34°C [23, 53]. As shown in Table 1, the gelation temperature increased when the concentration of poloxamer407 was decreased. The same phenomenon was found when the concentration of poloxamer188 was increased. When the concentration of poloxamer407 was ≥24% and the concentration of poloxamer188 was ≤2.0%, the gelation temperature of the formulation was <25°C. The formulation easily gelled during manufacturing and was unsuitable for administration. If the concentration of poloxamer407 was ≤ 18%, the gelation temperature of formulation was >34°C. The formulations couldn’t be used because they remained in liquid state and were easily washed away in nasal cavity. When the concentration of poloxamer407 was >18% and <24%, some gelation temperature was >34°C, too. The addition of poloxamer188 provided more alternative in the optimization of the formulation in the present study. The differential effects of P188 and P407 on the gelation temperature resulted from the different proportions of PPO and PEO subunits. As shown in Table 2, the addition of drug and additives had some effects on the gelation temperature, too. The gelation temperature decreased with the addition of the hydroxypropyl methylcellulose, because the hydroxypropyl methylcellulose could bind to polyethylene oxide chains in poloxamer molecules. This binding hindered the interactions between water and poloxamer molecules, promoted dehydration, and caused an increase in the entanglement of adjacent molecules with more extensive intermolecular hydrogen bonding [54, 55]. The gelation temperature decreased when sodium chloride was added. The decrease in the gelation temperature was possible due to the salting-out effect of NaCl on PEO segments. It was known that the cloud point of PEO surfactants decreased due to the salting out effects caused by Na^+^ and Cl^-^[56, 57]. However, the addition of geniposide increased the gelation temperature by its water solubility characteristics.

The response surface methodology is a kind of mathematical and statistical technique for designing experiments, building models, evaluating the relative significance of several independent variables, and determining the optimum conditions for desirable response [58–60]. In this study, the central composite design was employed for determining the optimum condition of the gelation temperature. A 3-factor, 5-level full factorial design was employed for the optimization of the mucoadhesive, thermoreversible *in situ* nasal. To evaluate the adequacy of the model, analysis of variance (ANOVA) was applied. The ANOVA of the empirical second-order polynomial indicated that the model was highly significant (Table 5). The response surface methodology played an important auxiliary role in optimizing the thermoreversible *in situ* nasal gel.

Mucoadhesive strength of the formulation is another important index [61]. Nasal mucociliary clearance decreased contact time and drug absorption by transporting the drug to the nasopharynx and then to the gastric intestinal tract. The mucoadhesive strength between 4000-6000dyn/cm^2^ was considered adequate [17, 62]. As shown in Table 7, the concentration of HPMC influenced the mucoadhesive strength greatly. As concentration of HPMC increased from 0.1% to 1.0%, there was a significant increase in mucoadhesive strength from 4560 dyn/cm^2^ to 1 0935 dyn/cm^2^.

The gel strength is another important criterion. The gel strength values between 25–50 sec were essential [15, 63]. As shown in Table 7, as concentration of HPMC increased from 0.1% to 1.0%, there was a significant increase in gel strength from 29 sec to 67 sec. The gel strength values of optimized formulations (S6, S7) were more than 50 sec, which meant the formulations were too stiff and would cause discomfort to the mucosal surface. Gel strength of the optimized formulations (S3, S4 and S5) was suitable.

The pH values of all the formulations were 6.3–6.5 which were in the nasal physiological pH range 4.5–6.5 (Table 7) [64].The drug and agents didn’t effects the pH of the formulations. The drug contents of all optimized formulations were checked and found in the range of 97–101% (Table 8).

The membraneless model was applied to study geniposide release from thermosensitive gel *in vitro*. In general, this model allowed the release medium to directly contact the gel surface and was closer to the *in vivo* condition [37–39, 65]. The in vitro release profiles of geniposide from the optimized formulations and the gel corrosion profiles were obtained simultaneously. The in vitro release data of geniposide were evaluated kinetically using mathematical models like the Zero-order, First-order, Higuchi and Weibull (Table 9). The best-fit model was the Zero-order model (*R*^2^ = 0.9935–0.9959). Release of geniposide was controlled by gel corrosion.

*Ex vivo* drug permeation could give more valuable informations about drug behavior in vivo [41–42, 66]. In this study, the cumulative release of geniposide from the optimized thermoreversible *in situ* nasal gel was 83.1–89.8% (Table 10). As shown in Table 11, the geniposide permeation kinetics model of the optimized formulations was fitted to the Weibull model (*R*^*2*^ = 0.9943–0.9979), which indicated the permeation of geniposide across nasal mucosa was possibly time-dependent. The more residence time of *in situ* nasal gel of geniposide was essential.

## Conclusions

The release of geniposide is controlled by gel corrosion. The permeation of geniposi- de is time dependent. The more residence time, mucoadhesive, thermoreversible *in situ* nasal gel of geniposide for neurodegenerative diseases is of compliance and potential application. The attractive *in situ* nasal gel of geniposide will be evaluated in further animal study.

## Supporting information

S1 Fig Cube plot of the effects of P408, P188 and HPMC on Tgel at a time. The predicted values from the coded model were P407 (19.2–22.8%), P188 (2.4–6.6%) and HPMC (0.3–0.8%).
(TIF)

S2 Fig The normal plot of residuals. Distribution of Tgel points indicates that the transformation of the response may provide a better analysis.
(TIF)

S1 Table Diagnostic statistics. The report of the residual, leverage, fitted value DFFITS and other statistics indicate that not all actual values are ideal and some are suitable. So the optimized formulations must be testified and achieve the suitable formulation.
(DOC)
