# Supplementary figures and images for: A mucoadhesive, thermoreversible in situ nasal gel of geniposide for neurodegenerative diseases
Source: PLoS One. 2017 Dec 14;12(12):e0189478. doi: 10.1371/journal.pone.0189478 (PMC5730156; doi:10.1371/journal.pone.0189478)

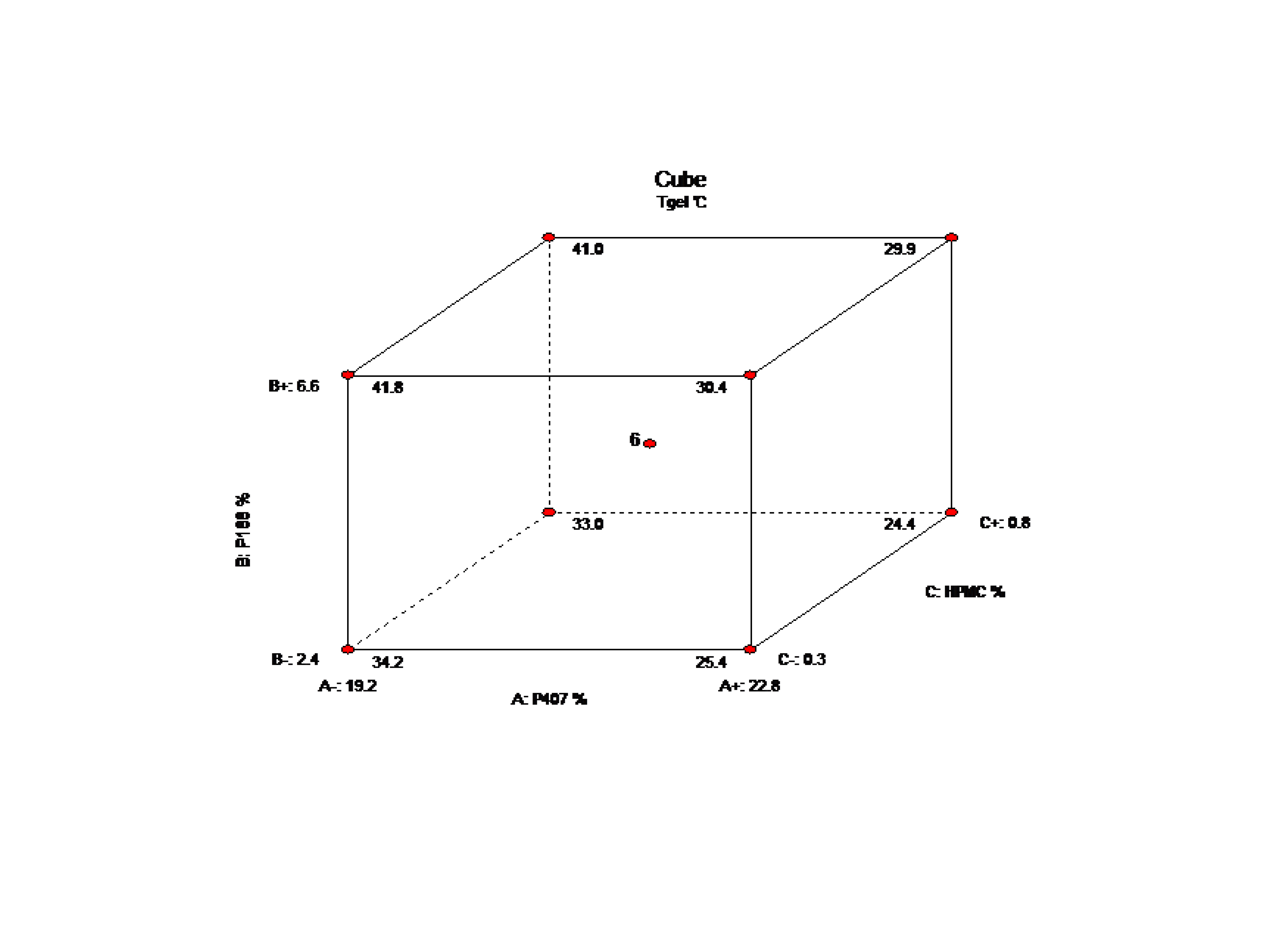

Supplement: S1 Fig — The predicted values from the coded model were P407 (19.2–22.8%), P188 (2.4–6.6%) and HPMC (0.3–0.8%). (TIF) [file pone.0189478.s001.tif]

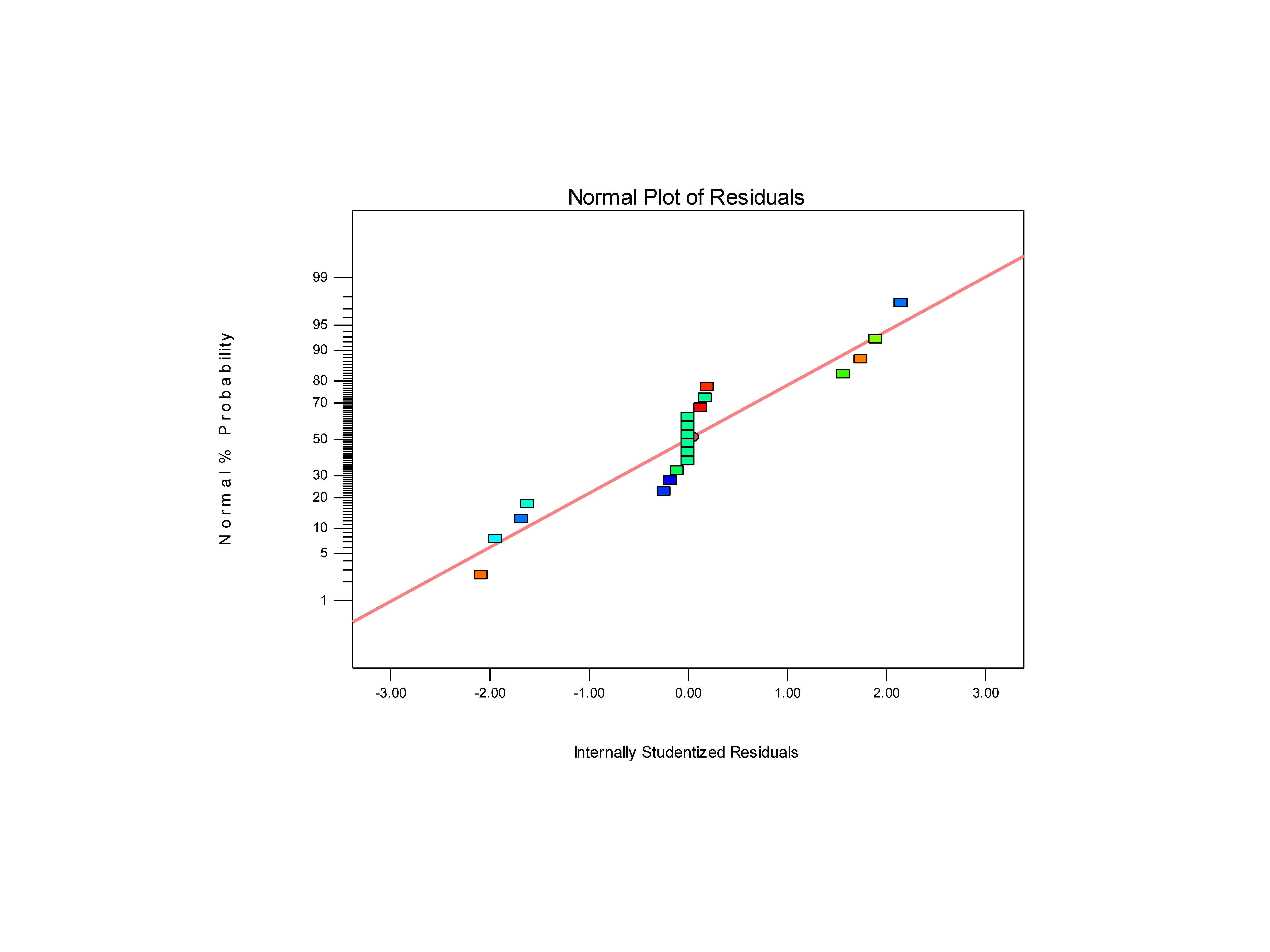

Supplement: S2 Fig — Distribution of Tgel points indicates that the transformation of the response may provide a better analysis. (TIF) [file pone.0189478.s002.tif]
