# Supplementary material for: A mucoadhesive, thermoreversible in situ nasal gel of geniposide for neurodegenerative diseases
Source: PLoS One. 2017 Dec 14;12(12):e0189478. doi: 10.1371/journal.pone.0189478 (PMC5730156; doi:10.1371/journal.pone.0189478)
Supplement: S1 Table — The report of the residual, leverage, fitted value DFFITS and other statistics indicate that not all actual values are ideal and some are suitable. So the optimized formulations must be testified and achieve the suitable formulation. (DOC) [file pone.0189478.s003.doc]

**S1_fig.tif Cube plot of the effects of P408, P188 and HPMC on Tgel at a time.** The predicted values from the coded model were P407 (19.2-22.8%), P188 (2.4-6.6%) and HPMC (0.3-0.8%).


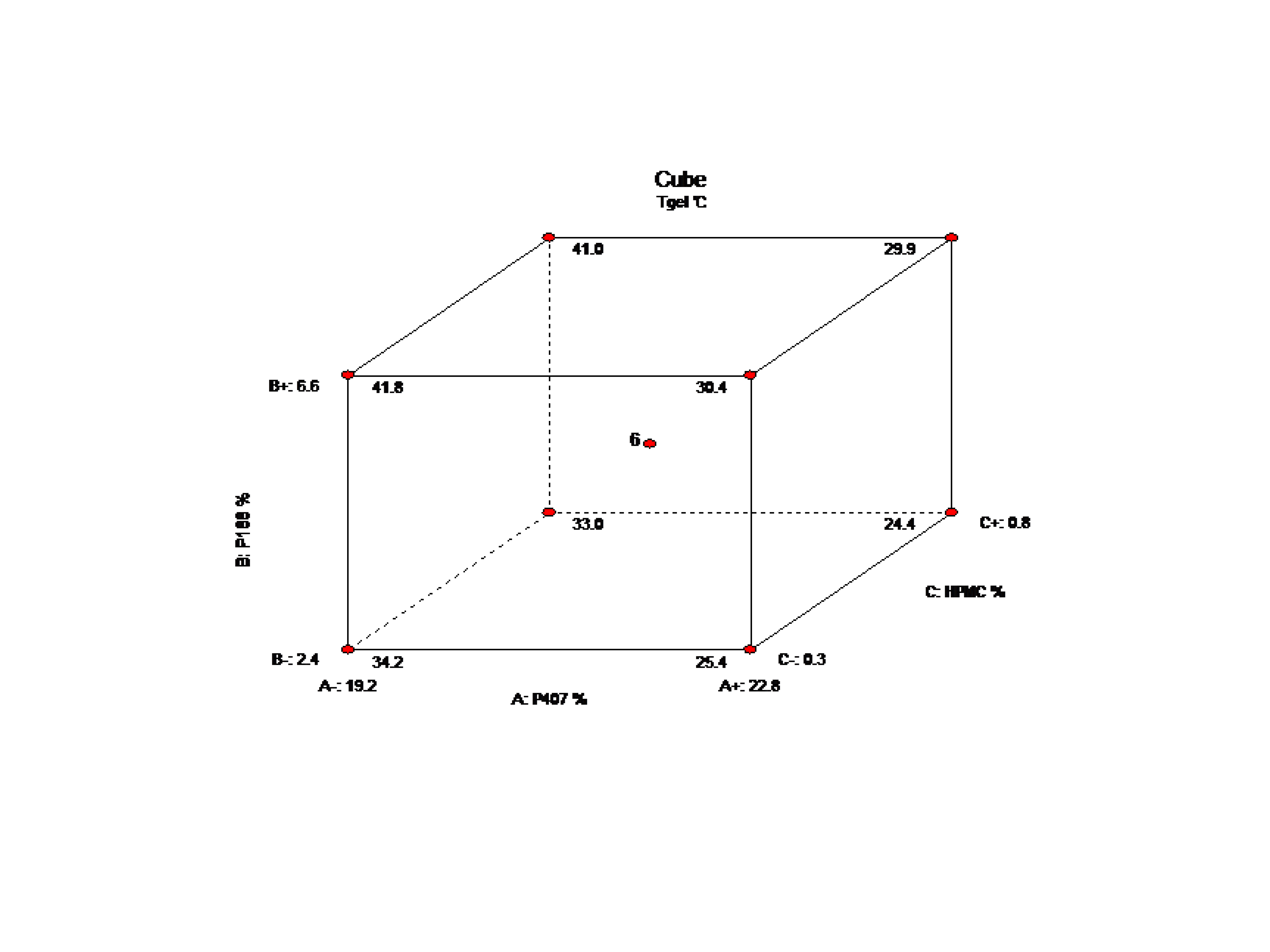


**S2_fig.tif The Normal plot of residuals**. Distribution of Tgel points indicates that the transformation of the response may provide a better analysis.


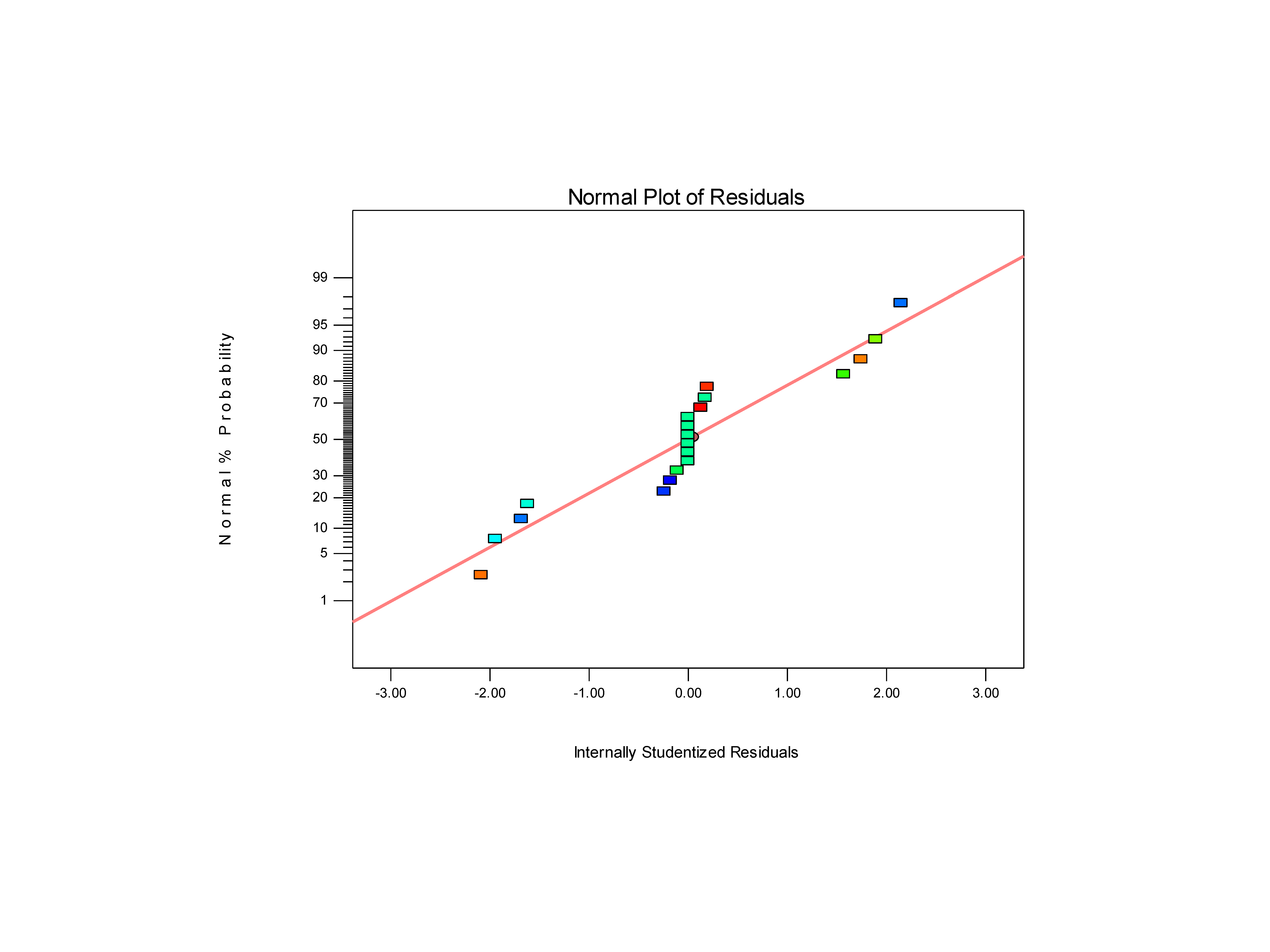


**S1 Table Diagnostic statistics.** The report of the residual, leverage, fitted value DFFITS and other statistics indicate that not all actual values are ideal and some are suitable. So the optimized formulations must be testified and achieve the suitable formulation.

|  | **Predicted**  **Value** | **Residual** | **Leverage** | **Internally Studentized**  **Residual** | **Externally Studentized**  **Residual** | **Influence on** | | **Run**  **Order** |
| --- | --- | --- | --- | --- | --- | --- | --- | --- |
|  |  |  |  |  |  | **Fitted Value DFFITS** | **Cook's Distance** |  |
| 35.4 | 34.18 | 1.22 | 0.67 | 1.894 | 2.243 | * 3.19 | 0.727 | 18 |
| 25.2 | 25.36 | -0.16 | 0.67 | -0.242 | -0.23 | -0.328 | 0.012 | 8 |
| 41.9 | 41.82 | 0.083 | 0.67 | 0.129 | 0.122 | 0.174 | 0.003 | 2 |
| 29.4 | 30.44 | -1.04 | 0.67 | -1.619 | -1.787 | * -2.55 | 0.531 | 1 |
| 34 | 32.99 | 1.01 | 0.67 | 1.57 | 1.715 | * 2.44 | 0.5 | 12 |
| 24.3 | 24.41 | -0.11 | 0.67 | -0.178 | -0.169 | -0.241 | 0.006 | 5 |
| 41.1 | 40.98 | 0.12 | 0.67 | 0.193 | 0.184 | 0.261 | 0.008 | 11 |
| 28.6 | 29.85 | -1.25 | 0.67 | -1.942 | -2.335 | * -3.33 | 0.765 | 9 |
| 40 | 41.47 | -1.47 | 0.607 | -2.086 | -2.632 | * -3.27 | 0.673 | 10 |
| 26.2 | 24.69 | 1.51 | 0.607 | 2.149 | 2.779 | * 3.46 | 0.714 | 13 |
| 26.3 | 27.48 | -1.18 | 0.607 | -1.681 | -1.883 | * -2.34 | 0.437 | 19 |
| 39.7 | 38.47 | 1.23 | 0.607 | 1.745 | 1.984 | * 2.47 | 0.471 | 17 |
| 31.8 | 31.88 | -0.077 | 0.607 | -0.11 | -0.104 | -0.13 | 0.002 | 3 |
| 30.5 | 30.38 | 0.12 | 0.607 | 0.173 | 0.165 | 0.205 | 0.005 | 7 |
| 30.6 | 30.6 | -1.27E-03 | 0.166 | -0.001 | -0.001 | -0.001 | 0 | 14 |
| 30.6 | 30.6 | -1.27E-03 | 0.166 | -0.001 | -0.001 | -0.001 | 0 | 6 |
| 30.6 | 30.6 | -1.27E-03 | 0.166 | -0.001 | -0.001 | -0.001 | 0 | 20 |
| 30.6 | 30.6 | -1.27E-03 | 0.166 | -0.001 | -0.001 | -0.001 | 0 | 16 |
| 30.6 | 30.6 | -1.27E-03 | 0.166 | -0.001 | -0.001 | -0.001 | 0 | 15 |
| 30.6 | 30.6 | -1.27E-03 | 0.166 | -0.001 | -0.001 | -0.001 | 0 | 4 |
